# Supplementary material for: Antibacterial and Osteogenic Properties of Ag Nanoparticles and Ag/TiO2 Nanostructures Prepared by Atomic Layer Deposition
Source: J Funct Biomater. 2022 May 18;13(2):62. doi: 10.3390/jfb13020062 (PMC9149969; doi:10.3390/jfb13020062)
Supplement: Supplementary file 1 [file jfb-13-00062-s001.zip › jfb-1695594-supplementary.pdf]

Supplementary Information

Antibacterial and Osteogenic Properties of Ag Nanoparticles and Ag/TiO_2 Nanostructures Prepared by Atomic Layer Deposition

Denis Nazarov [1,2,*], Ilya Ezhov [2], Natalia Yudintceva [3], Maxim Shevtsov [3,4], Aida Rudakova [1], Vladimir Kalganov [1], Vladimir Tolmachev [5], Yuliya Zharova [5], Oleksiy Lutakov [6], Ludmila Kraeva [7], Elizaveta Rogacheva [7] and Maxim Maximov [2]

[1] Saint Petersburg State University, Universitetskaya nab, 7/9, 199034 Saint Petersburg, Russia; aida.rudakova@spbu.ru (A.R.); vdkalganov@yandex.ru (V.K.)
[2] Peter the Great Saint Petersburg Polytechnic University, Polytechnicheskaya, 29, 195221 Saint Petersburg, Russia; iezhov1994@gmail.com (I.E.); maximspbstu@mail.ru (M.M.)
[3] Institute of Cytology of the Russian Academy of Sciences (RAS), Tikhoretsky Ave., 4, 194064 Saint Petersburg, Russia; yudintceva@mail.ru (N.Y.); shevtsov-max@mail.ru (M.S.)
[4] Center of Translational Cancer Research (TranslaTUM), Klinikum Rechts der Isar, Technical University Munich, Einstein Str. 25, 81675 Munich, Germany
[5] Ioffe Institute, Polytechnicheskaya, 26, 194021 Saint Petersburg, Russia; tva@mail.ioffe.ru (V.T.); piliouguina@mail.ioffe.ru (Y.Z.)
[6] Department of Solid State Engineering, Institute of Chemical Technology, 16628 Prague, Czech Republic; oleksiy.lyutakov@vscht.cz
[7] Saint-Petersburg Pasteur Institute of Epidemiology and Microbiology, 14 Mira Street, 197101 Saint Petersburg, Russia; lykraeva@yandex.ru (L.K.); elizvla@yandex.ru (E.R.)
* Correspondence: dennazar1@yandex.ru; Tel.: +7-812-428-4033

Table S1. The effect of Ag(fod)(PEt_3) evaporator temperature on the ALD silver growth.

Temperature of Ag(fod)(PEt_3) Evaporator, °C	Reactor Temperature, °C	Ag(fod)(PEt_3) Pulse Time/ Purge Time, s	Number of ALD Cycles	Thickness, nm	Growth Per Cycle, nm
130	147	4/5	350	<1	0.0014 ± 0.0014
140	156	4/5	700	1.9-3.9	0.0041 ± 0.0014
150	165	4/5	400	1.1-2.5	0.0045 ± 0.0018
160	173	4/5	400	0.3-1.1	0.0018 ± 0.001
170	173	4/5	400	0	0
180	182	4/5	350	0	0

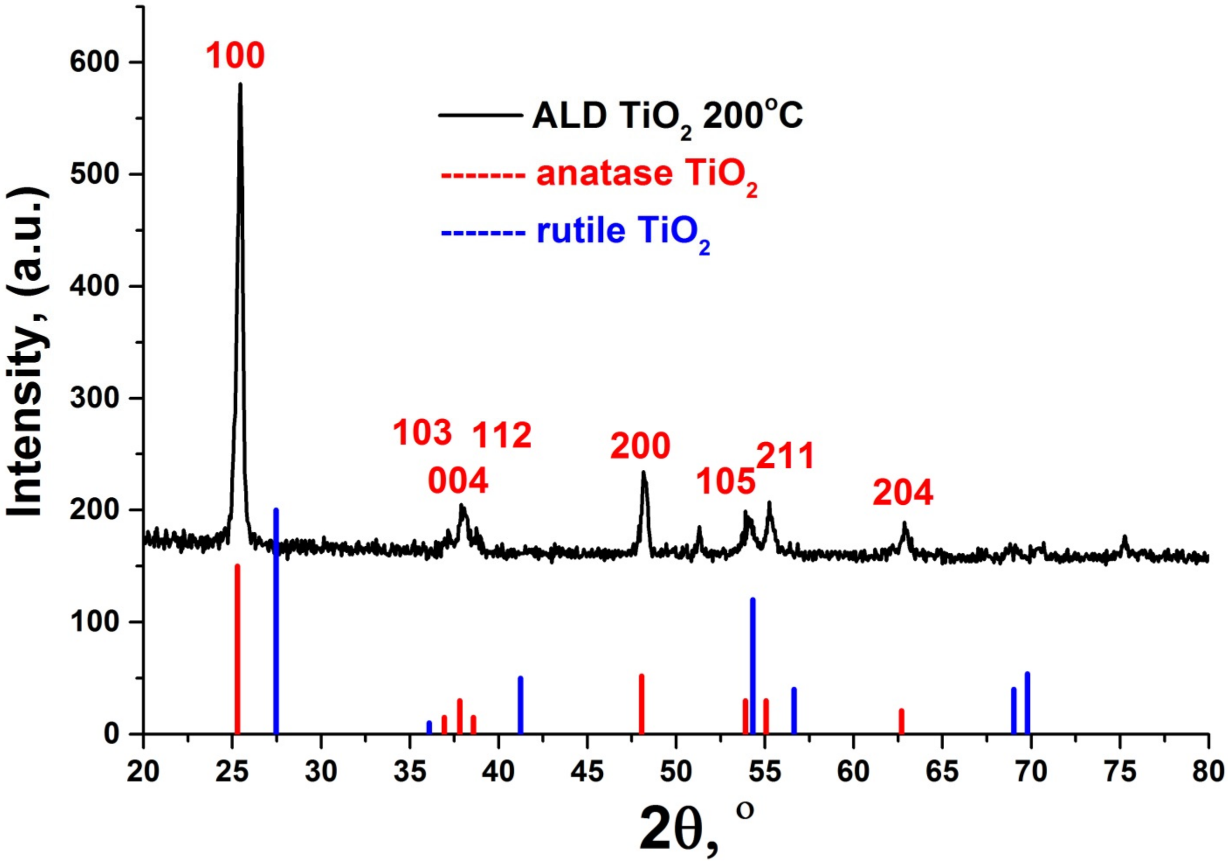

Figure S1. GIXRD pattern of the ALD TiO_2 nanolayers deposited on the silicon at 200 °C.

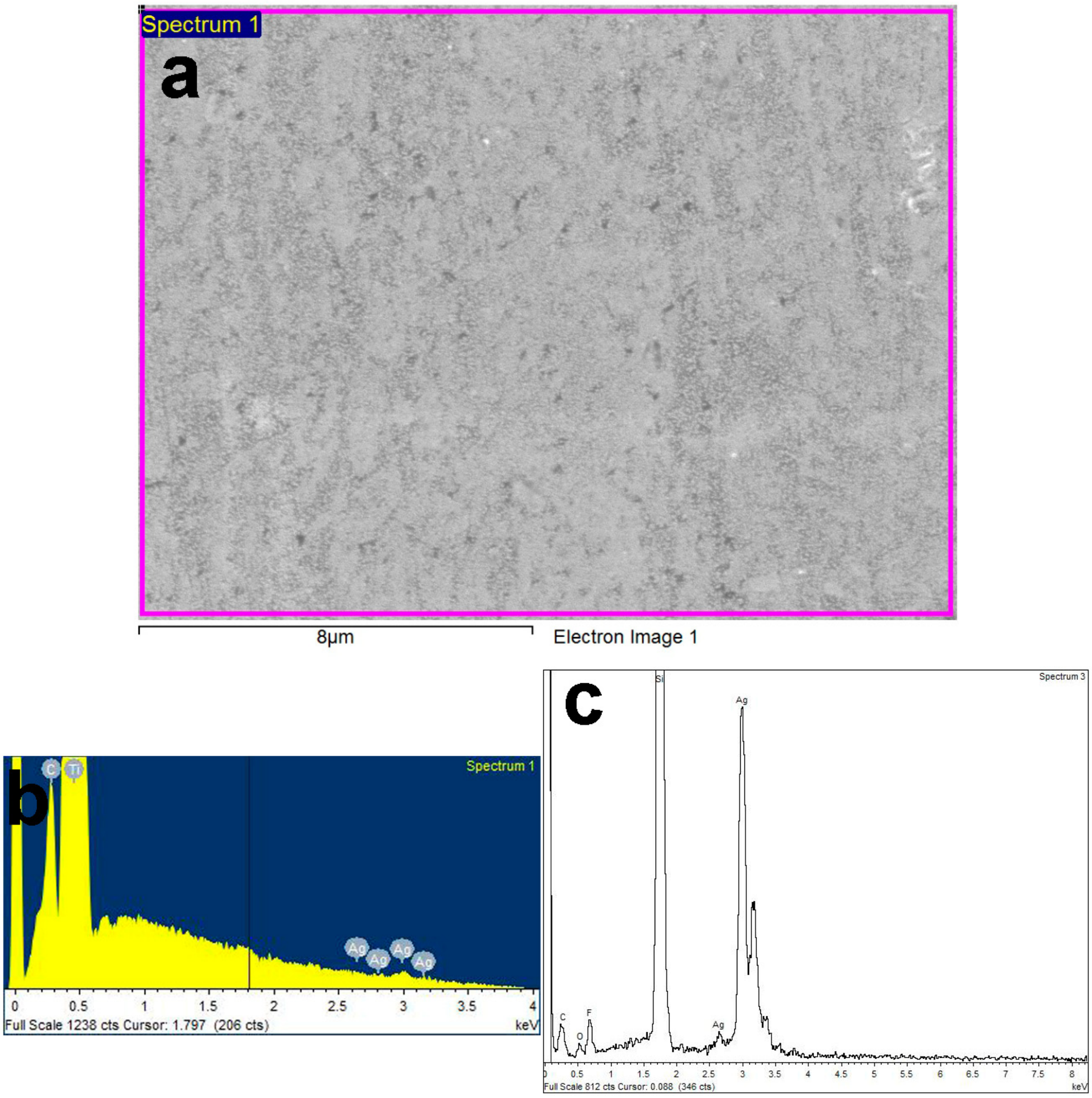

Figure S2. SEM-EDS results for Ti-Ag samples. SEM image of scan area for EDS measurements (**a**), EDS spectrum of full scan area (**b**), EDS spectrum of random point (**c**).

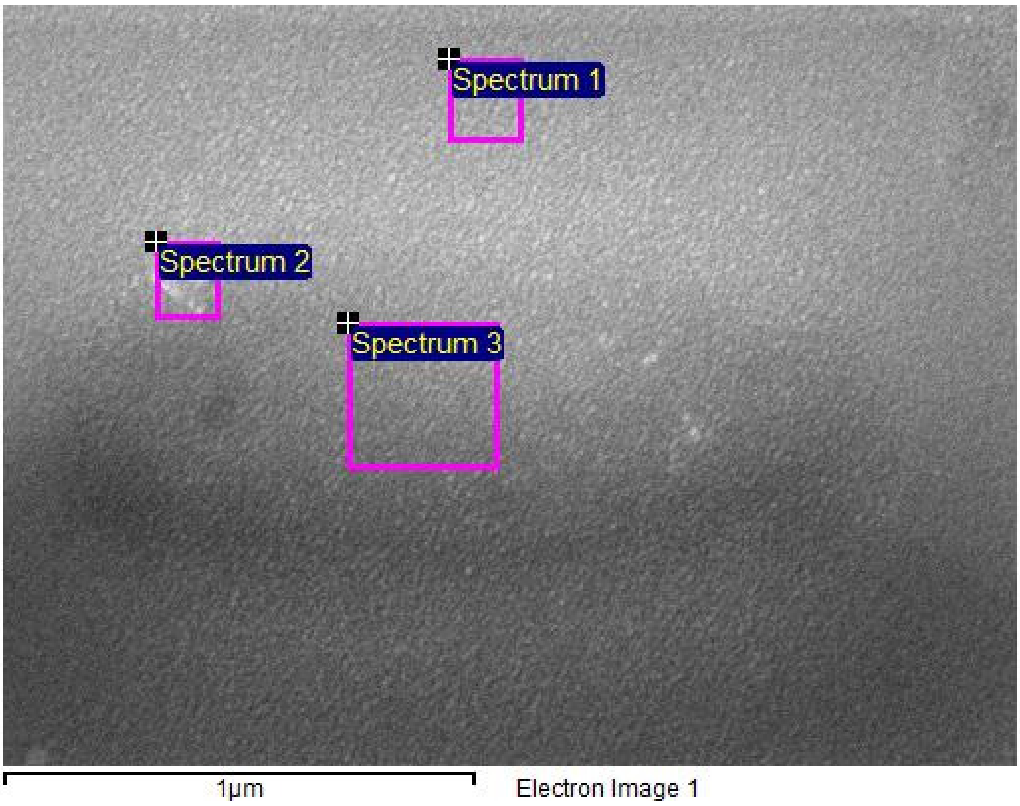

Figure S3. SEM image of scan area for EDS measurements of Ti-TiO_2-Ag sample.

Table S2. Results SEM-EDS study of Ti-TiO_2-Ag sample in different scan areas.

Spectrum	O, at %	Ti, at %	Ag, at %
Spectrum 1	7.08	92.33	0.58
Spectrum 2	9.22	89.96	0.82
Spectrum 3	7.77	91.68	0.55
Mean	8.02	91.32	0.65

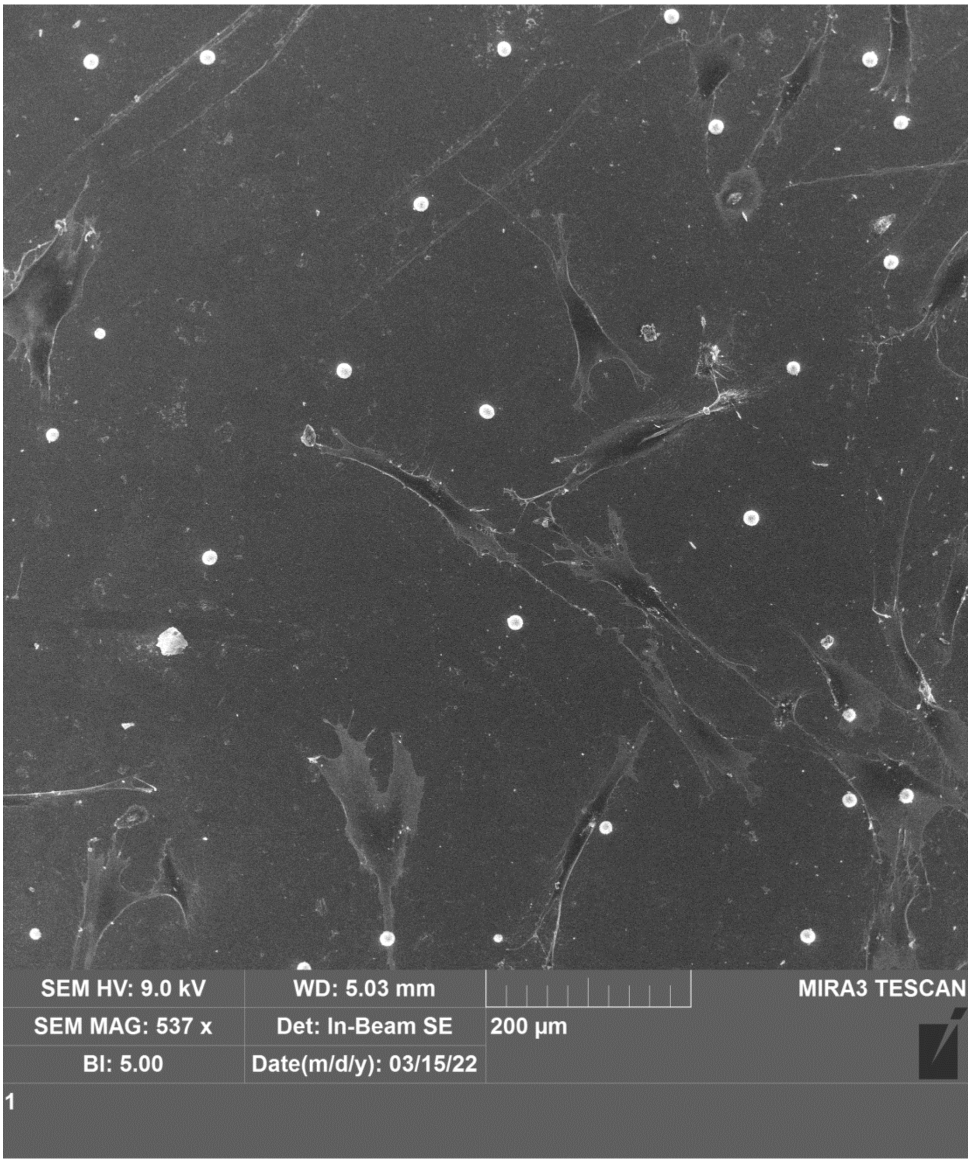

Figure S4. SEM image of FetMSCs cultivating during 24 h on the surface Ti-TiO_2 sample.
